# Minimally Interrupted Non-Vitamin K Antagonist Oral Anticoagulants vs. Bridging Therapy and Uninterrupted Vitamin K Antagonists During Atrial Fibrillation Ablation: A Retrospective Single-Center Study

**DOI:** 10.3389/fmed.2020.00197

**Published:** 2020-06-04

**Authors:** Lihong Tang, Haiyan Liu, Hai Deng, Xianzhang Zhan, Xianhong Fang, Hongtao Liao, Yang Liu, Lu Fu, Zuyi Fu, Huiyi Liu, Shulin Wu, Yumei Xue

**Affiliations:** ^1^Guangdong Cardiovascular Institute, Guangdong Provincial People's Hospital, Guangdong Academy of Medical Sciences, Guangzhou, China; ^2^Guangdong Provincial Key Laboratory of Clinical Pharmacology, Guangdong Provincial People's Hospital, Guangdong Academy of Medical Sciences, Guangzhou, China

**Keywords:** ablation, anticoagulation, atrial fibrillation, bleeding events, thromboembolism

## Abstract

**Objectives:** Although the latest international guidelines recommend the use of uninterrupted non-vitamin K antagonist oral anticoagulants (NOAC) during atrial fibrillation (AF) ablation, it does not reflect current clinical practice, as most centers still use a minimally interrupted NOAC strategy. The purpose of this study was to evaluate the safety and effectiveness of minimally interrupted NOAC compared with bridging therapy and uninterrupted vitamin K antagonist (VKA) for nonvalvular AF ablation.

**Patients and Methods:** A total of 4520 patients who underwent AF ablation between January 2010 and December 2018 were included in the analysis. According to their periprocedural anticoagulation strategies, patients were divided into three groups: Bridging heparin group (*n* = 1848); Uninterrupted VKA group (*n* = 796) and Minimally interrupted NOAC group (Total *n* = 1876; dabigatran: *n* = 865; rivaroxaban, *n* = 1011). A combined complication endpoint (CCE) as composed of any bleeding complications and thromboembolic events was analyzed.

**Results:** Rates of thromboembolisms were similar among the three groups (0.22% for Bridging heparin group, 0.25% for Uninterrupted VKA group, and 0.11% for Minimally interrupted NOAC group, *p* = 0.626). There was a significant difference among the three groups for the incidence of overall bleeding events (8.50% for Bridging heparin group, 4.52% for Uninterrupted VKA group, and 2.67% for Minimally interrupted NOAC group, *p* < 0.001). A significant difference of CCE rates was shown in the Minimally interrupted NOAC group as compared with the Uninterrupted VKA group (2.77 vs. 4.77%, *p* = 0.008) and the Bridging heparin group (2.77 vs. 8.71%, *p* < 0.001). There was no significant difference in CCE rates among the different NOACs (dabigatran 2.89% vs. rivaroxaban 2.67%, *p* = 0.773).

**Conclusions:** In patients undergoing AF ablation, minimally interrupted NOACs during the periprocedural period appears safer and equally effective when compared to the bridging heparin and uninterrupted VKA therapy.

## Introduction

Catheter ablation is a safe, effective, and promising strategy for patients with symptomatic atrial fibrillation (AF). Careful attention to periprocedural anticoagulation for AF ablation is mandatory to reduce the risk of major complications comprising stroke, transient ischemic attack (TIA), and cardiac tamponade (1). Currently, it is a well-established anticoagulation strategy that uninterrupted warfarin therapy is associated with a lower risk of periprocedural bleeding and strokes than stopping vitamin K antagonist (VKA) and bridging with heparin (2). However, in an era in which non-vitamin K antagonist oral anticoagulants (NOACs) are increasingly used, recent randomized controlled trials (RCTs) have demonstrated that uninterrupted NOAC is as safe and effective in comparison to uninterrupted VKA in patients undergoing AF ablation (3–6). The 2017 Consensus Statement on Catheter and Surgical Ablation of AF give a class I or IIa indication to perform AF ablation without withholding the NOACs and VKAs or withholding one to two doses of the NOACs prior to ablation (1).

Recent meta-analysis reported a similar incidence of thromboembolic events and lower incidence of bleeding in patients with either interrupted or uninterrupted NOACs therapy as compared with uninterrupted VKAs therapy (7–9). However, the major concern in performing AF ablation with uninterrupted NOACs is the risk of bleeding, particularly life-threatening bleeding such as pericardial tamponade. At present, the uninterrupted NOAC strategy does not reflect current clinical practice, as most centers still use a minimally interrupted NOAC strategy (10). The aim of the present study was to evaluate the safety and effectiveness of minimally interrupted NOAC compared with bridging therapy and uninterrupted VKA during AF ablation in a “real-world” clinical practice.

## Patients and Methods

### Study Population

Consecutive patients who underwent catheter ablation of AF from January 2010 to December 2018 were recorded. The inclusion criteria were as follows: (1) adult patients (≥ 18 years of age); (2) patients undergoing either radiofrequency ablation or cryoablation for AF. The exclusion criteria were as follows: (1) presence of valvular heart disease; (2) diagnosis of malignant tumors; and (3) incomplete follow-up data within 30 days postablation.

Depending on the periprocedural anticoagulation strategies prescribed by prior physicians, patients were divided into three groups. The first group included patients who discontinued warfarin and bridging with heparin. The second group included patients who used periprocedural uninterrupted VKA. The third group included patients who used periprocedural minimally interrupted NOAC (Dabigatran: 110 mg twice daily; Rivaroxaban: 15 or 10 mg once daily). A transesophageal echocardiography (TEE) was routinely performed on the same day or 1 day before ablation to exclude left atrial appendage (LAA) thrombus. Computed tomography (CT) scans were performed only in a minority of patients who were intolerant to TEE.

The studies involving human participants were reviewed and approved by the Clinical Research Ethics Committee of Guangdong Provincial People's Hospital. The patients provided their written informed consent to participate in this study.

### Periprocedural Protocol

In the Bridging heparin group, warfarin was usually withheld for 3–5 days, and enoxaparin 1 mg/kg every 12 h was given until the evening before the ablation procedure with a target international normalized ratio (INR) < 2. In the Uninterrupted VKA group, with patients using continued warfarin, the target INR level at the day of the procedure was 2–3. In the Minimally interrupted NOAC group, the last dabigatran was given in the evening on the day before the procedure, while rivaroxaban was given on the morning of the day before the procedure. On the morning of the procedure NOACs were paused. Neither low molecular weight heparin (LMWH) nor unfractionated heparin was administered during the preprocedural period in both the Uninterrupted VKA group and Minimally interrupted NOAC group.

During the procedure, intravenous heparin (100 U/kg) was given immediately after transseptal puncture and adjusted to maintain a target activated clotting time (ACT) of 250–350 s. Protamine was not routinely used at the end of the procedure.

As for the Bridging heparin group, enoxaparin 0.5 mg/kg twice daily was used as a bridge to resumption of warfarin therapy until INR ≥ 2. In patients treated with uninterrupted VKA who had an INR 2.0 or greater on the day of the procedure, warfarin was continued the evening of the procedure with a target INR level of 2–3. In patients treated with minimally interrupted NOAC preablation dabigatran or rivaroxaban was restarted 3–5 h after ablation. No LMWH or unfractionated heparin was administered in both the Uninterrupted VKA group and Minimally interrupted NOAC group after ablation. All patients were continued on anticoagulation for at least 2 months postablation.

### Definitions

Any bleeding or thromboembolic event during the procedure and within 30 days postablation was categorized. Bleeding complications were defined by the Bleeding Academic Research Consortium (BARC) and International Society on Thrombosis and Haemostasis (ISTH) (11, 12). Accordingly, major bleeding events included cardiac tamponade or pericardial effusions requiring drainage, intracranial and major gastrointestinal hemorrhages, a hemothorax, retroperitoneal bleeding, any bleeding requiring a blood transfusion, and vascular access site complications requiring any intervention. Bleeding events that did not fulfill the ISTH criteria for major bleeding were considered nonmajor bleeding events. Thromboembolic events were defined as the occurrence of symptomatic stroke/transient ischemic attack (TIA), peripheral embolic events, or deep venous thrombosis. However, no routine postablation brain magnetic resonance imaging (MRI) was performed to test for “silent” cerebral events. Furthermore, we defined a combined complication endpoint (CCE) as composed of any bleeding complications and thromboembolic events.

### Statistical Analysis

Continuous variables were presented as mean ± SD and compared using one-way analysis of variance (ANOVA) test. Categorical variables were presented as counts and percentages and compared using the χ^2^ test or Fisher exact test as appropriate. Multivariable logistic regression was used to identify significant predictors of CCE. All potential confounders were entered into the model on the basis of known clinical relevance or significant association observed in univariate analysis. The adjusted odds ratio and 95% confidence interval (CI) were computed.

Four confounders (age, type of AF, CHA_2_DS_2_-VASc score, and HAS-BLED score) were adjusted for unequal patient characteristics due to nonrandomization. After that, a Kaplan–Meier analysis and log-rank test were used to compare the CCE rates within 30 days after ablation procedure among groups. A *p*-value < 0.05 was considered statistically significant. All statistical analysis was performed using SPSS software (version 24.0, IBM, Armonk, NY, USA).

## Results

**Figure 1:**
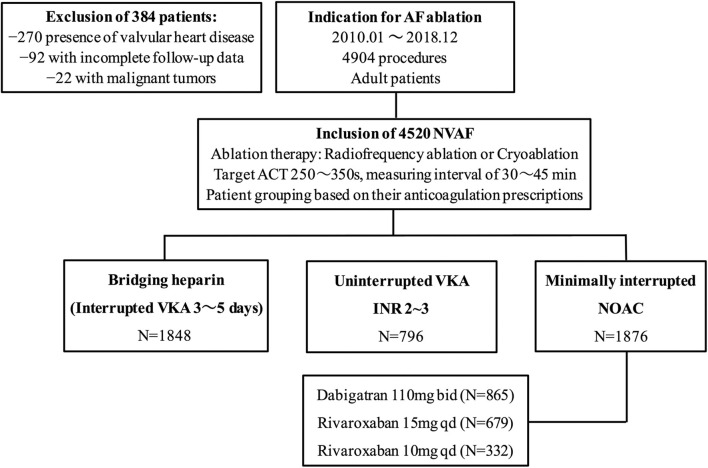
Study flow chart. AF, atrial fibrillation; NVAF, nonvalvular atrial fibrillation; ACT, activated clotting time; VKA, vitamin K antagonist; NOAC, non-vitamin K antagonist oral anticoagulant.

**Figure 2:**
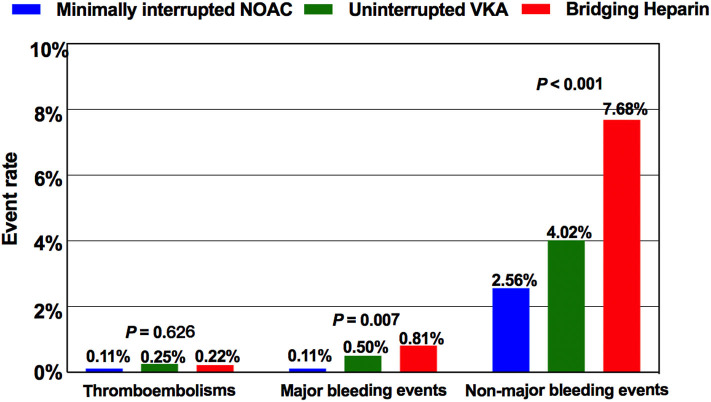
Comparison of the incidence of thromboembolisms and bleeding events among the three anticoagulation protocols. NOAC, non-vitamin K antagonist oral anticoagulant; VKA, vitamin K antagonist.

Of a total of 4,904 patients who underwent AF ablation, 384 were excluded due to the coexistence of valvular heart disease, malignant tumors, and incomplete follow-up data within 30 days postablation. Among the remaining 4520 patients, AF ablation was performed with a “Bridging heparin” protocol in 1848 patients (40.9%), an “Uninterrupted VKA” protocol in 796 patients (17.6%), and a “Minimally interrupted NOACs” protocol in 1876 patients (41.5%) (Figure 1). The majority of patients were male (65.9%), and the average age was 58 years. The median CHA_2_DS_2_-VASc score was 1, and a CHA_2_DS_2_-VASc ≥ 2 accounted for 43.3% of the cohort with no significant difference among the three groups (*p* = 0.255). Patients had a median HAS-BLED of 1 (range 0–4), with the majority being classed as 0 or 1 across groups and 2.7% having a score ≥ 3. There were significant differences among groups with respect to their bleeding risk (*p* = 0.009). Baseline characteristics of the three groups are summarized in Table 1. Furthermore, the incidence of thromboembolisms and bleeding events among the three anticoagulation strategies are shown in Figure 2.

**Table 1:** Baseline characteristics.

	**Bridging heparin**	**Uninterrupted VKA**	**Minimally interrupted NOAC**	***p-*value**
	**(*N* = 1848)**	**(*N* = 796)**	**(*N* = 1876)**	
Age, years	57 ± 11	60 ± 11	59 ± 11	<0.001
Male, *n* (%)	1227 (66.4)	511 (64.2)	1239 (66.0)	0.537
Persistent AF, *n* (%)	386 (20.9)	167 (21.0)	377 (20.1)	0.753
Hypertension, *n* (%)	758 (41.0)	310 (38.9)	731 (39.0)	0.381
Diabetes mellitus, n (%)	234 (12.7)	109 (13.7)	227 (12.1)	0.523
CAD, *n* (%)	272 (14.7)	118 (14.8)	309 (16.5)	0.288
Vascular disease, *n* (%)	203 (11.0)	73 (9.2)	231 (12.3)	0.058
Heart failure, *n* (%)	50 (2.7)	25 (3.1)	55 (2.9)	0.814
Prior stroke/TIA, *n* (%)	114 (6.2)	52 (6.5)	124 (6.6)	0.851
Renal dysfunction, *n* (%)	38 (2.1)	23 (2.9)	27 (1.4)	0.042
CHA_2_DS_2_-VASc ≥ 2, *n* (%)	776 (42.0)	360 (45.2)	823 (43.9)	0.255
HAS-BLED ≥ 3, *n* (%)	33 (1.8)	26 (3.3)	62 (3.3)	0.009

*Values are given as the mean ± SD or n (%)*.

*CAD, coronary artery disease; CHA_2_DS_2_-VASc score, congestive heart failure, hypertension, diabetes mellitus, vascular disease, an age of 65–74 years, female sex (1 point for each), age ≥75 years, stroke/TIA (2 points for each); HAS-BLED, hypertension, abnormal renal and liver function, a stroke, bleeding history/predisposition, labile international normalized ratio, age >65 years, drugs/alcohol concomitantly (1 point for each); NOAC, non-vitamin K antagonist oral anticoagulant; TIA, transient ischemic attack; VKA, vitamin K antagonist*.

### Bleeding Complications

In our study, 243 overall bleeding events were reported; the overall event rate was 8.50% in patients receiving bridging regimen with LMWH, 4.52% in patients receiving uninterrupted warfarin, and 2.67% in patients receiving minimally interrupted NOACs (Table 2). There was significant difference among the three groups for the incidence of overall bleeding events (*p* < 0.001). The number of major bleeding events was similar between the Minimally interrupted NOACs group and the Uninterrupted warfarin group (0.11 vs. 0.50%, OR 0.21; 95% CI 0.04–1.16; *p* = 0.073), while a lower trend was observed for the comparison between the Minimally interrupted NOACs group and the Bridging Heparin group (0.11 vs. 0.81%, OR 0.36; 95% CI 0.17–0.76; *p* = 0.007).

**Table 2:** Details on bleeding and thromboembolic complication rates.

	**Bridging heparin**	**Uninterrupted VKA**	**Minimally interrupted NOAC**
Major bleedings			
Pericardial tamponade	15 (0.81)	4 (0.50)	2 (0.11)
Other major bleedings^a^	0	0	0
Total	15 (0.81)	4 (0.50)	2 (0.11)
Non-major bleedings			
Pericardial effusion not requiring drainage	5 (0.27)	0	1 (0.05)
Groin bleeding/hematoma	72 (3.90)	19 (2.39)	26 (1.39)
Femoral pseudoaneurysm	5 (0.27)	1 (0.13)	0
Femoral arteriovenous fistula	0	1 (0.13)	3 (0.16)
Other non-major bleedings^b^	60 (3.25)	11 (1.38)	18 (0.96)
Total	142 (7.68)	32 (4.02)	48 (2.56)
Thromboembolic complications			
Stroke	3 (0.16)	2 (0.25)	2 (0.11)
Deep venous thrombosis	1 (0.05)	0	0
Total	4 (0.22)	2 (0.25)	2 (0.11)
Combined bleedings total	157 (8.50)	36 (4.52)	50 (2.67)
Combined complication endpoint	161 (8.71)	38 (4.77)	52 (2.77)

*Values are presented as n (%)*.

*NOAC, non-vitamin K antagonist oral anticoagulant; VKA, vitamin K antagonist*.

a *Other major bleedings including any bleeding event that required a blood transfusion and/or intervention*.

b *Other non-major bleedings including hematuria, epistaxis, ecchymosis, and minor gastrointestinal bleeding*.

In a further breakdown of major bleeding, there were a total of 21 pericardial tamponades requiring drainage (total incidence 0.46%; 15 in the Bridging heparin group, 4 in the Warfarin group, and 2 in the NOAC group, Table 3). Among them, a majority of the events (17/21, 81.0%) occurred during the procedure, while others occurred within 6 hours after the procedure. Emergency percutaneous pericardiocentesis with subxyphoid access was performed in all 21 patients. The mean drained blood volumes were 686 mL. Protamine was administered in 14 patients for periprocedural heparin reversal. Direct autologous blood transfusion (AutoBT) was performed routinely as salvage therapy to allow maintenance the hemodynamic status (13). Despite pericardiocentesis, anticoagulation reversal, and AutoBT, an emergent open-chest surgery was required in 11 (52.4%) patients due to persistent reaccumulation of blood in the pericardial space. Most of the patients completely recovered without any deficit while one patient died of cardiorespiratory arrest without surgery. In addition, no other major bleeding events were recorded.

**Table 3:** Clinical details of the pericardial tamponade.

**Pericardial tamponade (*N* = 21)**	
Female, *n* (%)	6 (28.6)
Age (years), mean ± SD	66 ± 8
Anticoagulation strategy, *n* (%)	
Bridging heparin	15 (71.4)
Uninterrupted warfarin	4 (19.0)
Minimally interrupted NOACs	2 (9.5)
Ablation techniques, *n* (%)	
RFCA	19 (90.5)
Cryo	2 (9.5)
Time of event onset, *n* (%)	
Intraprocedure	17 (81.0)
Within 6 h postprocedure	4 (19.0)
Pericardiocentesis, *n* (%)	21 (100)
Drained blood volume (mL), mean ± SD	686 ± 463
AutoBT, *n* (%)	15 (71.4)
Protamine used, *n* (%)	14 (66.7)
Surgey, *n* (%)	11 (52.4)
Death, *n* (%)	1 (4.8)

*Values are given as the mean ± SD or n (%)*.

*AutoBT, autologous blood transfusion; Cryo, cryoablation; NOAC, non-vitamin K antagonist oral anticoagulant; RFCA, radiofrequency ablation*.

Non-major bleeding complications were the most frequent bleeding complications overall, led by minor groin complications (127/243; 52.3%) not requiring any intervention treatment. Most of the minor groin complications (including groin bleeding/hematoma, arteriovenous fistula, and pseudoaneurysm) occurred in the first 3 days after ablation procedure. In addition to the vascular access complications mentioned earlier, the occurrence times of other non-major bleeding events were irregular. Patients in the Minimally interrupted NOACs group had a lower risk of suffering non-major bleeding events in comparison to both the Uninterrupted warfarin group (OR 0.63; 95% CI 0.40–0.99; *p* = 0.044) and the Bridging heparin group (OR 0.56; 95% CI 0.48–0.66; *p* < 0.001). More details about non-major bleeding events are shown in Table 2.

### Thromboembolic Complications

The incidence of thromboembolic events was low (Bridging heparin, 0.22%; Uninterrupted VKA, 0.25%; Minimally interrupted NOAC, 0.11%) and not significantly different among groups (*p* = 0.626). Of the 8 thromboembolic complications documented, 7 (87.5%; 2 in the Bridging heparin group, 3 in Uninterrupted VKA group, and 2 in the Minimally interrupted NOAC group) were ischemic stroke events. Clinical details of the 7 patients with stroke events are shown in Table 4. Most complications (5/7, 71.4%) occurred either during the procedure or within 1 day postablation. At the time of stroke events, more than one half of the patients were in sinus rhythm. Continuous anticoagulation, receiving catheter ablation, and/or left atrial appendage closure might not guarantee that the stroke events would be avoided. CT or MRI scan revealed lesions were usually located in the cerebellar hemisphere, basal ganglia, temporal lobe, and right thalamus. All patients received conservative treatments except one patient who was treated by using emergent stent retriever thrombectomy 2 h after stoke onset. Furthermore, one patient in the Bridging heparin group experienced deep venous thrombosis 5 days after the procedure. Finally, he had an uneventful recovery after conservative treatments.

**Table 4:** Patients, characteristics, and management of stroke complications.

**Case**	**Age/**	**CHA_**2**_DS_**2**_-VAS_**C**_**	**Anticoagulant**	**Ablation**	**Time^a^**	**Rhythm^a^**	**Lesion location**	**Management**
	**sex**							
1	61/F	3	Bridge	RFCA	Intraoperative	SR	Right cerebellar hemisphere	Conservative treatments
2	71/F	6	Bridge	RFCA	Intraoperative	SR	Left basal ganglia	CAG and thrombectomy
3	74/M	5	Bridge	RFCA	1 d postoperative	AF	Left temporal lobe and cerebellar hemisphere	Conservative treatments
4	48/M	0	Uninterrupted warfarin	RFCA	1 d postoperative	AF	Right thalamus	Conservative treatments
5	70/M	1	Uninterrupted warfarin	RFCA	7 d postoperative	SR	Right basal ganglia and temporal lobe	CAG and conservative treatments
6	69/M	3	NOAC (rivaroxaban)	Cryo+LAAC	1 m postoperative	AF	Right basal ganglia	Conservative treatments
7	48/F	1	NOAC (dabigatran)	RFCA	1 d postoperative	SR	Left frontal lobe and temporal lobe	Conservative treatments

*CAG, cerebral angiography; Cryo, cryoablation; LAAC, left atrial appendage closure*.

*NOAC, non-vitamin K antagonist oral anticoagulant; RFCA, radiofrequency ablation; SR, sinus rhythm*.

a *Time and rhythm were recorded when the event occu0rred*.

### Combined Complication Endpoint and Subgroup Analysis

Patients were divided into two groups according to CCE outcome status, and univariate analysis was performed to compare their clinical characteristics (Table 5). Age, male gender, prior stroke/TIA, renal dysfunction, CHA_2_DS_2_-VASc ≥ 2, HAS-BLED ≥ 3, and anticoagulation strategies were found to be significantly associated with occurrence of CCE.

**Table 5:** Univariate and multivariate analysis of clinical variables affecting CCE.

	**Total no**.	**CCE**	**Univariate analysis**		**Multivariate analysis (Model 1)**		**Multivariate analysis (Model 2)**	
		**no. (%)**	**OR (95% CI)**	***p*-value**	**OR (95% CI)**	***p*-value**	**OR (95% CI)**	***p*-value**
Age, years			1.026 (1.014–1.039)	<0.001	1.028 (1.015–1.042)	<0.001		
Male	2977	151 (5.1)	1.297 (0.999–1.683)	0.050	1.199 (0.916–1.569)	0.186		
Persistent AF	930	52 (5.6)	1.009 (0.737–1.382)	0.954	1.084 (0.786–1.495)	0.624	1.489 (1.144–1.938)	0.938
Hypertension	1799	112 (6.2)	1.233 (0.954–1.594)	0.109	1.012 (0.774–1.324)	0.929		
Diabetes mellitus	570	30 (5.3)	0.937 (0.633–1.387)	0.746	0.804 (0.538–1.199)	0.284		
CAD	699	46 (6.6)	1.243 (0.893–1.729)	0.198	1.128 (0.803–1.583)	0.488		
Vascular disease	507	34 (6.7)	1.257 (0.865–1.828)	0.230	1.175 (0.801–1.723)	0.411		
Heart failure	130	10 (7.7)	1.435 (0.743–2.770)	0.282	1.349 (0.686–2.650)	0.386		
Prior stroke/TIA	290	24 (8.3)	1.261 (1.013–1.571)	0.038	1.202 (0.960–1.506)	0.109		
Renal dysfunction	88	12 (13.6)	2.770 (1.486–5.163)	0.001	2.485 (1.309–4.718)	0.005		
CHA_2_DS_2_-VASc ≥ 2	1959	134 (6.8)	1.534 (1.188–1.980)	0.001			1.488 (1.143–1.937)	0.003
HAS-BLED ≥ 3	121	14 (11.6)	2.298 (1.297–4.072)	0.004			2.235 (1.230–4.060)	0.008
Anticoagulation strategy				<0.001		<0.001		<0.001
Minimally interrupted NOACs	1876	52 (2.8)	Reference		Reference		Reference	
Uninterrupted VKA^a^	796	38 (4.8)	1.758 (1.148–2.695)	0.010	1.708 (1.111–2.624)	0.015	1.755 (1.144–2.692)	0.010
Bridging heparin^b^	1848	161 (8.7)	3.348 (2.432–4.608)	<0.001	3.539 (2.564–4.885)	<0.001	3.462 (2.511–4.773)	<0.001

*CCE, combined complication endpoint; CAD, coronary artery disease; NOAC, non-vitamin K antagonist oral anticoagulant; TIA, transient ischemic attack; VKA, vitamin K antagonist*.

a *Uninterrupted VKA : uninterrupted VKA vs. minimally interrupted NOAC*.

b *Bridging heparin: bridging heparin vs. minimally interrupted NOAC*.

Subsequently, a multivariable analysis was performed with the logistic regression model (model 1). With the exception of CHA_2_DS_2_-VASc ≥ 2 and HAS-BLED ≥ 3, the significant confounders identified in the univariable test were included in the model. Besides, some other clinically important covariates were added to the model despite their nonsignificant association in the univariate test. After adjustment for these confounders, it was found that the age, renal dysfunction, and anticoagulation strategies were independent risk factors of CCE. To avoid multicollinearity, another multivariable model was run by including AF type, CHA_2_DS_2_-VASc ≥ 2, HAS-BLED ≥ 3, and anticoagulation strategies (model 2). As observed in this model, all the aforementioned confounders were independently associated with CCE except the type of AF. In both models, the minimally interrupted NOAC strategy was considered the reference and was compared with the uninterrupted VKA strategy and bridging heparin strategy. The odds ratios from these two models are presented in Table 5.

**Figure 3:**
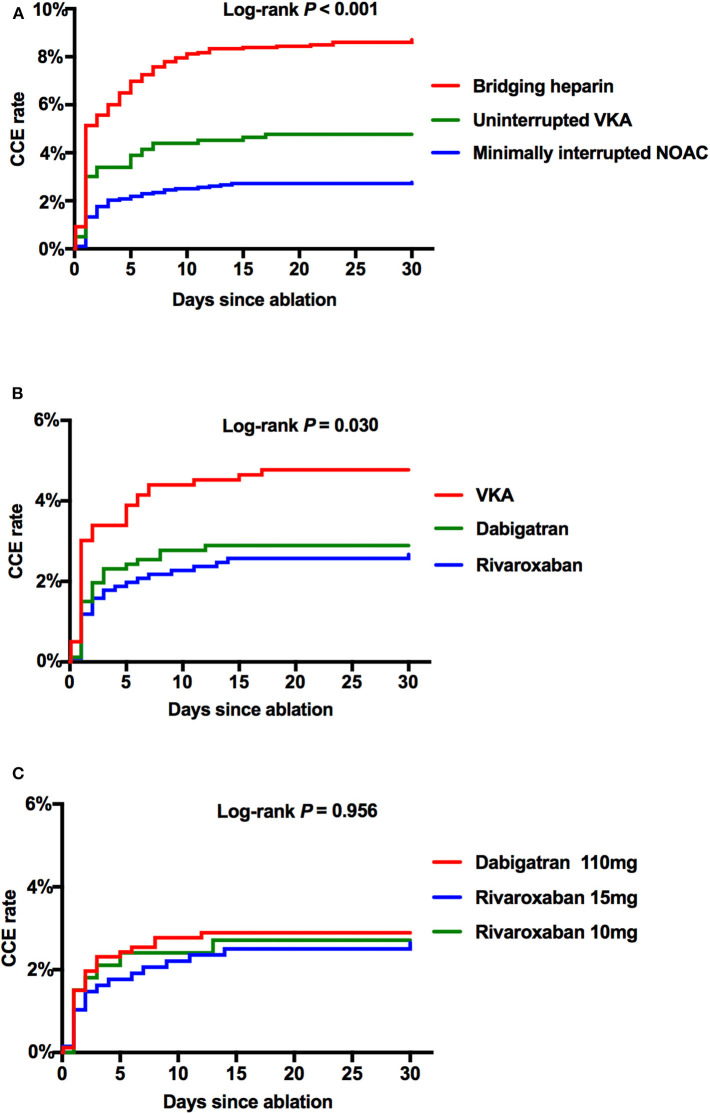
The Kaplan–Meier curves showing the event rates of CCE after the ablation procedure. **(A)** Comparison among Bridging, VKA, and NOAC. **(B)** Comparison among VKA, dabigatran 110 mg, and total rivaroxaban. **(C)** Comparison among dabigatran 110 mg, rivaroxaban 15 mg, and rivaroxaban 10 mg. CCE, combined complication endpoint; NOAC, non-vitamin K antagonist oral anticoagulant; VKA, vitamin K antagonist.

Cumulative event rates of CCE between subgroups within 30 days after the ablation procedure were shown by a Kaplan–Meier analysis. After adjusted by age, AF type, CHA_2_DS_2_-VASc, and HAS-BLED score, a significant difference of CCE rates were shown in the Minimally interrupted NOAC group as compared with the Uninterrupted VKA group (2.77 vs. 4.77%, *p* = 0.008) and the Bridging heparin group (2.77 vs. 8.71%, *p* < 0.001) (Figure 3A). Further subgroup analyses were performed between the VKA group and individual NOAC groups. The incidence rate of CCE was similar between the dabigatran group and the rivaroxaban group (2.89 vs. 2.67%, *p* = 0.773), and a similar trend was observed for the comparison between the rivaroxaban 15 mg and 10 mg groups (2.65 vs. 2.71%, *p* = 0.956) (Figures 3B,C).

## Discussion

We retrospectively evaluated the safety and effectiveness of minimally interrupted NOAC compared with bridging therapy and uninterrupted VKA for nonvalvulare AF ablation in a real-world setting. Furthermore, NOACs were also compared individually with uninterrupted VKAs in our study. The main findings were as follows: (1) the risk of thromboembolism in the current anticoagulant regimens is exceedingly rare and not significantly different between groups; (2) minimally interrupted NOACs are associated with a lower incidence of bleeding complications in comparison with uninterrupted warfarin and bridging therapy; (3) the incidence of CCE was similar between the dabigatran and rivaroxaban groups.

Great advances have been made in periprocedural anticoagulation therapy for patients with AF ablation during the past few years. In the warfarin era, continuous warfarin therapy during AF ablation reduced the risks of thromboembolic and bleeding complications as compared with a bridging regimen with LMWH (2). This uninterrupted warfarin strategy had gained wider acceptance and was endorsed in the 2012 expert consensus statement and 2014 AHA/ACC/HRS guideline (14, 15). With the emergence of NOACs, increasing numbers of patients with AF are on NOACs during the catheter ablation due to its advantages of rapid onset of therapeutic anticoagulation, shorter duration of action, and relatively fewer drug interactions. In recent years, several meta-analyses and RCTs favored performing AF ablation with uninterrupted NOACs, as this strategy has been linked to a similar incidence of thromboembolic events and lower incidence of bleeding complications compared to uninterrupted warfarin therapy (3–9). However, an antidote is not commercially available for NOACs (except for dabigatran), and the existing published data about reversal agents from experienced centers are limited. The major concern of uninterrupted NOACs might be the risk of major bleeding during ablation, particularly in Asian populations, who have a higher risk of bleeding than persons of other races (16). Therefore, a minimally interrupted NOAC during AF ablation as an alternative to uninterrupted NOACs strategy in our center seems to be reasonable, which also gained a IIa indication in the 2017 expert consensus statement (1).

While previous studies had demonstrated less major bleeding with continues NOAC versus VKA, this NOAC dosing strategy does not reflect widespread current clinical practice (9). The European Snapshot Survey on Procedural Routines in Atrial Fibrillation Ablation (ESS-PRAFA) in 2015 demonstrated that only 14% of patients were on an NOAC uninterrupted therapy (10). However, a majority of the patients (53%) underwent the procedure with a minimally interrupted NOAC strategy (one or two doses withheld), followed by 34% of the patients on interrupted NOAC ≥ 2 days. As compared with the uninterrupted warfarin strategy, the ABRIDGE-J trial showed that minimally interrupted dabigatran (one or two doses withheld) was associated with fewer major bleeding events without compromising thromboembolic safety (17). In addition, results from the Apixaban Evaluation of Interrupted Or Uninterrupted anticoagulation for ablation of atrial fibrillation (AEIOU) randomized trial showed no difference between continuous apixaban and minimally interrupted apixaban (one dose withheld) for the risk of major bleeding and thromboembolic events (18). Furthermore, a recent meta-analysis including 13 studies (4 randomized and 9 observational) with 5463 patients found that minimally interrupted and continuous NOAC strategy were both safe and non-inferior periprocedural strategies in comparison to uninterrupted VKA (19). Given the current lack of standardized protocols for the periprocedural management of minimally interrupted NOAC and interrupted or uninterrupted VKA therapy still a common strategy (20), our study based on a modest proportional shift away from bridging and VKA toward NOAC use over time which reflecting a real-world practice of periprocedural anticoagulant therapy. Furthermore, we found a similar low complication rates in patients receiving dabigatran as compared to rivaroxaban. There was no statistical difference with regard to the incidence rates of CCE between different NOAC groups.

Previous studies reported the incidence of periprocedural thromboembolic events in patients undergoing AF ablation ranges from 0.1 to 1.1% (21, 22), and bleeding complications were occurred within a range of 12–20% (23). In our study, the overall rate of thromboembolic events was 0.22% in the Bridging heparin group, 0.25% in the Uninterrupted VKA group, and 0.11% in the Minimally interrupted NOAC group. The overall rate of bleeding complications was 8.50% in the Bridging heparin group, 4.52% in the Uninterrupted VKA group, and 2.67% in the Minimally interrupted NOAC group. This is comparable, albeit lower than in previously studies. Currently, one of the most important reasons to choose an uninterrupted NOAC or VKA strategy instead of a minimally interrupted NOAC therapy is to minimize the incidence of periprocedural thromboembolic events. However, we found a 50% relative risk reduction in thromboembolic complications with minimally interrupted NOACs (0.11%) as compared with uninterrupted VKA (0.25%), although the single endpoint missed statistical significance. It should be emphasized that uninterrupted NOAC or VKA does not prevent all acute brain lesion, which can be caused by air embolism or debris from ablation lesions (24). Furthermore, a low event rates of thromboembolism might result in a limited statistical power to delineate differences among groups.

As for major bleeding, a similar low incidence was noted (Minimally interrupted NOAC 0.11% vs. Uninterrupted VKA 0.50%) while no significant difference was detected (*p* = 0.073). It should be considered that pericardial tamponade—the only major bleeding events in our study—is mostly caused by technical factors. It is important to note that it is not the anticoagulant that causes spontaneous bleeding, but the inherent risk of the procedure. A misdirected transseptal puncture, direct mechanical trauma, and overheating during the radiofrequency energy delivery are the most common causes leading to cardiac tamponade (14). Recently, a study focused on the management of cardiac tamponade in AF ablation had revealed there was no significant difference between the anticoagulant used with the occurrence of events (25), which was consistent with our findings.

Previous meta-analysis reported a reduced incidence of non-major bleeding when withholding several does of NOACs before the procedure (8). In our study, patients in the minimally interrupted NOACs group had a lower risk of suffering non-major bleeding events both in comparison to the Uninterrupted warfarin group (*p* = 0.044) and the Bridging Heparin group (*p* < 0.001). Patient-related bleeding risk was very low in our study because 97% of the patients had a HAS-BLED < 3. As the minor groin complications (including groin bleeding/hematoma, arteriovenous fistula, and pseudoaneurysm) were the most frequent non-major bleeding complications related to the technical factors, the influence of periprocedural anticoagulation on the complications is rather negligible. It should be noted that increasing numbers of patients with AF are on NOACs during the catheter ablation in the past 3 years, while bridging therapy and uninterrupted VKA were mostly used in our center before 2016. With the accumulation of procedure experience, this may lead to a decrease in ablation-related bleeding complications.

Undoubtedly, there are some limitations in our observational study. First, the low incidence of events likely represented a population of highly experienced electrophysiologists performing the catheter ablation, which could have reduced the sensitivity of the results. Second, given that the grouping was retrospective based on medical records, a modest proportional shift away from bridging and VKA toward NOAC use over time was seen. As a result, there was a bias caused by historical changes in periprocedural anticoagulation strategies. Third, no routine MRI was performed to detect asymptomatic cranial lesions following the ablation, which might have underestimated the incidence of stroke. Fourth, this study was conducted in Chinese patients; thus, the findings cannot be generalized to other ethnic populations. Despite these limitations, our study was carried out under the real-world use of periprocedural anticoagulation strategy in AF ablation during the past few years, which might provide some valuable insights into clinical practice.

## Conclusions

In patients undergoing AF ablation, minimally interrupted NOACs during the periprocedural period appears safer and equally effective compared to the bridging heparin and uninterrupted VKA therapy. Both minimally interrupted dabigatran or rivaroxaban (one dose withheld) showed a non-inferior and even superior combined complication outcome compared to uninterrupted warfarin during the periprocedural period of the AF ablation.

## Data Availability Statement

The datasets generated for this study are available on request to the corresponding author.

## Ethics Statement

The studies involving human participants were reviewed and approved by Clinical Research Ethics Committee of Guangdong Provincial People's Hospital. The patients/participants provided their written informed consent to participate in this study. Written informed consent was obtained from the individual(s) for the publication of any potentially identifiable images or data included in this article.

## Author Contributions

LT, HaL, HD, SW, and YX contributed conception and design of the study. LT, HaL, XZ, XF, HL, and YL organized the database. LF, ZF, and HuL performed the statistical analysis. LT and HaL wrote the first draft of the manuscript. LT, HaL, HD, and YX wrote sections of the manuscript. All authors contributed to the manuscript revision and read and approved the submitted version.

## Conflict of Interest

The authors declare that the research was conducted in the absence of any commercial or financial relationships that could be construed as a potential conflict of interest.
